# Meta-Analysis of Pharmacokinetic Studies of Nanobiomaterials for the Prediction of Excretion Depending on Particle Characteristics

**DOI:** 10.3389/fbioe.2019.00405

**Published:** 2019-12-17

**Authors:** Marina Hauser, Bernd Nowack

**Affiliations:** Empa, Swiss Federal Laboratories for Materials Science and Technology, St. Gallen, Switzerland

**Keywords:** nanobiomaterials, pharmacokinetic, meta-analysis, excretion, prediction

## Abstract

The growth in development and use of nanobiomaterials (NBMs) has raised questions regarding their possible distribution in the environment. Because most NBMs are not yet available on the market and exposure monitoring is thus not possible, prospective exposure modeling is the method of choice to get information on their future environmental exposure. An important input for such models is the fraction of the NBM excreted after their application to humans. The aim of this study was to analyze the current literature on excretion of NBMs using a meta-analysis. Published pharmacokinetic data from *in vivo* animal experiments was collected and compiled in a database, including information on the material characteristics. An evaluation of the data showed that there is no correlation between the excretion (in % of injected dose, ID) and the material type, the dose, the zeta potential or the size of the particles. However, the excretion is dependent on the type of administration with orally administered NBMs being excreted to a larger extent than intravenously administered ones. A statistically significant difference was found for IV vs. oral and oral vs. inhalation. The database provided by this work can be used for future studies to parameterize the transfer of NBMs from humans to wastewater. Generic probability distributions of excretion for oral and IV-administration are provided to enable excretion modeling of NBMs without data for a specific NBM.

## Introduction

In the past decade, nanobiomaterials (NBMs) have been increasingly investigated for the use in pharmaceutics and biomedical engineering (Küster and Adler, 2014). A wide range of different nanomaterials are being suggested for these purposes. For example, metals or metal oxides are very common in nanomedicine. Their relatively simple generation and surface modification as well as biocompatibility make gold (Au) nanoparticles attractive for the utilization in medical imaging or cancer detection and treatment (Hirn et al., 2011; Bonakdar and Mashinchian, 2015; Rambanapasi et al., 2015). Silver (Ag) nanoparticles are applied as coatings for indwelling catheters, antibacterial agents, wound dressing, orthopedic implants, and tissue-engineered scaffolds (Lin et al., 2015). Silica nanoparticles (SiO_2_) are easy to synthesize, exhibit low toxicity and have an ease for surface modification. These properties make silica applicable as biomarkers, biosensors, DNA or drug delivery, and cancer therapy (Lee et al., 2014). Also organic nanomaterials are often used in medical applications, especially due to their high biological safety, good biodegradability, low environmental toxicity (Hauser et al., 2019), and easy production and modification (Han et al., 2018). Commonly used organic NBMs are chitosan, polylactic acid (PLA), or poly(lactic-glycolic acid) (PLGA). They may be preferred to other types of nanoparticles due to their flexibility, biodegradability, and relatively low levels of toxicity (Navarro et al., 2017). Chitosan is a polysaccharide which is found in the exoskeleton of crustaceans and is applied in fast wound healing or as a blood clotting agent (Singh et al., 2017). PLA is used in cartilage regeneration, bone tissue engineering, and cartilage repair due to its good elastic modulus, thermal formability, and mechanical strength. PLGA is widely used in nanoparticles, microspheres, pellets, sutures, implantable scaffolds, and microcapsules (Navarro et al., 2017; Han et al., 2018). Additionally, also carbon-based nanomaterials are used in nanomedicine. Fullerenes and carbon nanotubes (CNTs) are highly promising for medical applications as carriers in drug delivery (Yamashita et al., 2012).

NBMs can be administered to the patient's body in different ways. The most commonly used routes of administration in humans are oral, intravenous and inhalation. From these, the oral route is the most convenient one as it is non-invasive and therefore widely accepted by most patients (Schleh et al., 2012). Besides, it also has the potential to be taken at home and not necessarily in a hospital or clinic setting (Navarro et al., 2017). However, the absorption into the bloodstream after oral absorption is generally very low (Park et al., 2011; Lin et al., 2015). The lungs are considered the most important entry of nanoparticles into the human body for example via occupational inhalation of airborne particles during manufacturing (Li X. et al., 2012; Laux et al., 2017). The advantage of intravenous injection is the direct access of the NBM to the blood circulation and thereby a quick distribution throughout the entire body (Hirn et al., 2011). In animal studies also intratracheal (introduction of the material directly into the trachea) or intraperitoneal (into the body cavity) administration is common.

Increasing applications and usage of NBMs leads to an increase in the potential for environmental exposure (Laux et al., 2017; Kabir et al., 2018). Depending on the material, a NBM can biodegrade, accumulate in tissues and organs or get excreted via urine or feces. From urine and feces, they enter the sewage system and are eventually discharged into surface water from where they are distributed throughout the whole biosphere. We expect NBMs to behave similarly to pharmaceuticals as they have the same mode of application and are also excreted in urine and feces from where they reach the sewage system. The German Federal Environment Agency reported the detection of 156 pharmaceuticals in environmental media such as surface water, groundwater and drinking water (Umwelt Bundesamt, 2018). Pharmaceuticals were detected in surface water at a concentration of 0.1–10.0 μg/l (Bergmann et al., 2011).

In order to be able to assess the environmental exposure, one needs knowledge of the presence of nanomaterials in different products but also about their release throughout the life cycle (Som et al., 2010; Keller et al., 2013). The release of nanomaterials into the environment has previously been modeled for a range of engineered nanomaterials (Mueller and Nowack, 2008; Gottschalk et al., 2009, 2010; Sun et al., 2014, 2016, 2017; Wang et al., 2016). However, only one modeling study has been published for NBMs, covering the environmental exposure of gold-nanoparticles from medical applications in the United States and the United Kingdom (Mahapatra et al., 2015).

In exposure modeling the whole life cycle of the material needs to be taken into consideration. For NBMs, the excretion of the NBM from the body is the starting point from where they flow to the sewage system, the waste water treatment plant and finally can be distributed throughout the biosphere to reach different environmental compartments such as soil, ground water, oceans, as well as the atmosphere. In recent years, the number of published physiologically based pharmacokinetic models (PBPK) of NBMs has increased significantly (Grass and Sinko, 2002; Li et al., 2010; Li M. et al., 2012, 2016; Moss and Siccardi, 2014; Carlander et al., 2016; Li D. et al., 2016). These studies are mostly interested in the distribution of the NBMs in the body to different organs and tissues but the excretion of the material in feces or urine is in many cases also considered.

The aim of our study was to collect data from published pharmacokinetic studies of NBMs and make predictions based on this data set about the excretion of the NBM from the body. As different studies used different materials, coatings, administrations, doses, animals, and evaluation time spans, we aimed to incorporate the different materials and particle properties or study designs into the evaluation and to make general predictions about the excretion of NBMs.

## Methods

The literature was searched for pharmacokinetic studies of NBM or nanoparticles in general that specifically quantified excretion of the nanoparticles. The time frame of the search includes all studies until the end of April 2019. Google Scholar was used with search terms such as “pharmacokinetics nanoparticles excretion,” or “pharmacokinetics nanomaterials excretion,” “pharmacokinetics metallic/polymeric/organic/etc. nanoparticles/nanomaterials excretion” in all variations, or just “nanoparticles excretion.” For each search term, the first ten pages each containing 10 articles were looked at. Besides, the cited articles of these studies were also evaluated.

Only studies with a time frame of a least 1 day were considered. As we were only interested in the total excretion of the nanoparticles, studies with a time frame of <1 day were deemed too short to fully excrete the nanoparticles. Additionally, only studies where the excretion in feces and/or urine is mentioned in %ID (percent of injected dose) or total excretion with the amount administered mentioned in the article (so the %ID could be calculated) were considered. Within one study, only the data point at the longest time was collected per material as it was assumed that this shows the total excretion. Only one data point was collected per material per study to avoid overrepresentation of studies with many measurements. However, several data points were collected from one study if materials with different size, zeta potential, surface coating, dose, etc. were used.

For each material, the material class, the particle size (TEM measurements), the test animal, the route of administration, the zeta potential of the material, the administered dose, and the cumulative excretion (in %ID) were noted. We have taken these material characteristics as they were mentioned in other articles to be of significance for the excretion of the material (Soo Choi et al., 2007; Semmler-Behnke et al., 2008; Alric et al., 2013; Xu et al., 2018). TEM measurements of the primary particle size were preferred over hydrodynamic size as TEM measurements were more widely available and as the nanoparticles get rapidly modified by protein adsorption after administration in the body (Kreyling et al., 2014).

## Results and Discussion

### Presentation of the Database

**Figure 1:**
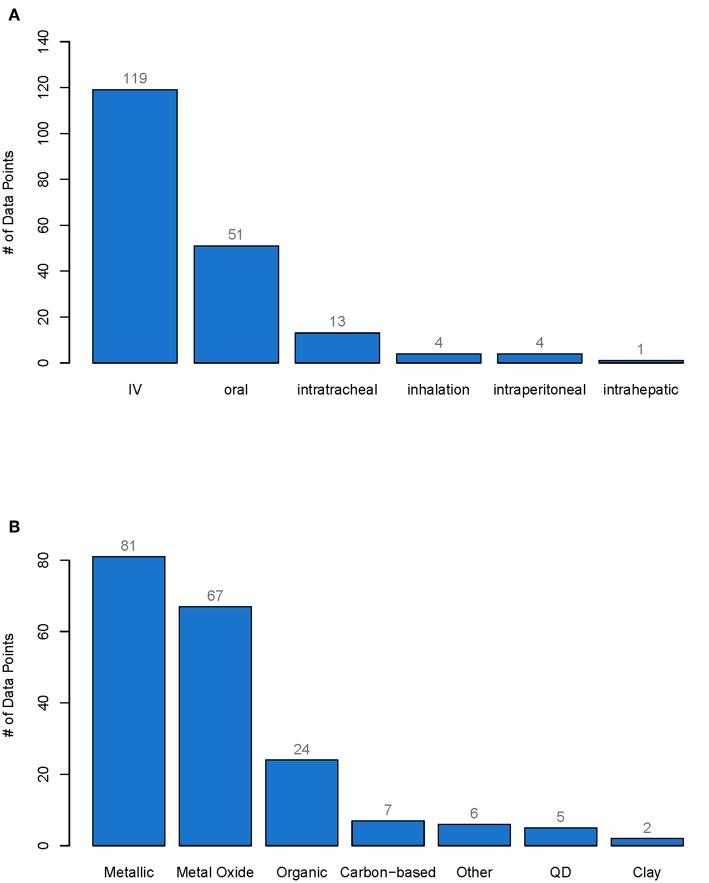
Number of data points for each type of administration **(A)** and for each type of material class **(B)**. From each pharmacokinetic study of nanobiomaterials only one data point was extracted per specific material and the cumulative excretion as well as the material properties were reported. The whole database with all data points can be found in the Supporting Information. IV, Intravenously administered, QD, Quantum dots.

In total, 192 data points were collected from 66 studies. The whole database can be found in the Table S1. More than 60% of the nanomaterials were administered intravenously (IV), 30% orally, 7% intratracheally, and <3% by inhalation or intraperitoneal or intrahepatic injection (see Figure 1A). Of all the materials investigated, 40% were metallic, 35% metal oxides, 12% organic and <4% carbon-based, Quantum Dots (QD), clays or other (see Figure 1B).

**Figure 2:**
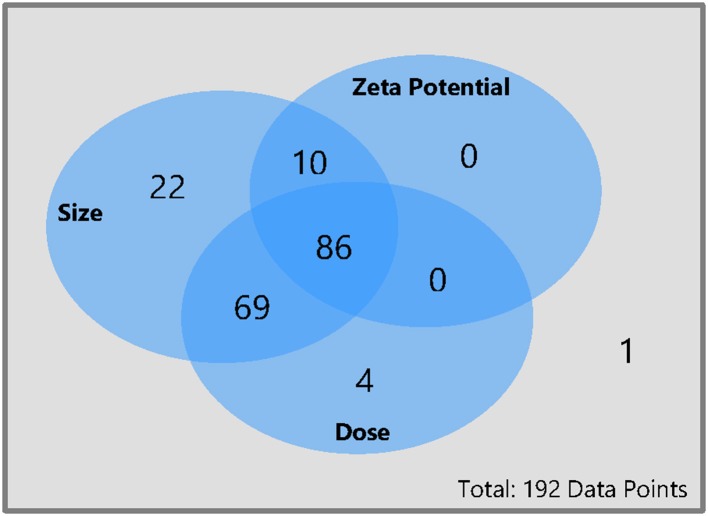
Availability of size, dose, and zeta potential for all data points collected for this meta-analysis. From each pharmacokinetic study of nanobiomaterials only one data point was extracted per specific material and the cumulative excretion as well as the material properties were reported. The whole database with all data points can be found in the Supporting Information.

Not all studies reported all relevant material or study characteristics. For almost 45% of the data points, the full data set with zeta potential, size, and administered dose was available (86 data points), see Figure 2. For six data points the zeta potential was only listed as positive or negative. These data points were counted as only size and dose available. For 36% of the data points only the size and the dose were mentioned but not the zeta potential, whereas for 5% of the data points only the size and the zeta potential was available but not the dose. For more than 10% of the data points only the size and for 2% of the data points only the dose could be found. For one data point, neither the size nor the dose or the zeta potential was mentioned in the article (see Figure 2). The particles ranged in size from 1.1 to 360 nm, the zeta potential ranged from −76 to 106.2 mV, and the administered dose ranged from 0.0032 to 2,000 mg/kg body weight.

**Figure 3:**
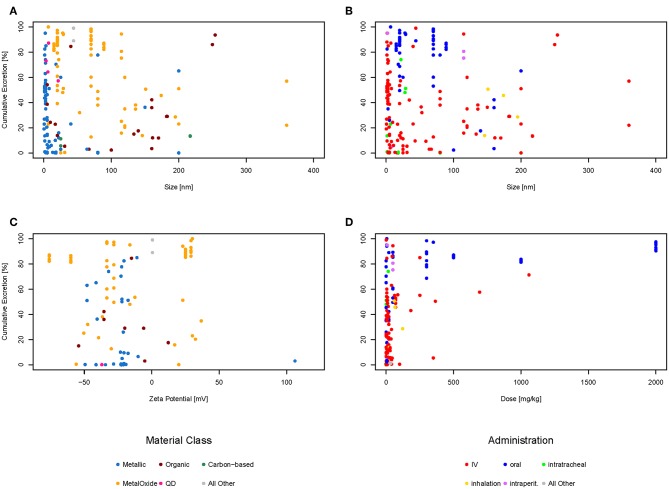
Cumulative excretion vs. size of nanoparticle color-coded by material class **(A)**, cumulative excretion vs. size of nanoparticle color-coded by type of administration **(B)**, cumulative excretion vs. zeta potential of nanoparticle color-coded by material class **(C)**, and cumulative excretion vs. dose of nanoparticle color-coded by type of administration **(D)**.

The amounts excreted through urine and feces were added together to get the total excretion of the nanomaterial. In order to evaluate if there is a relationship between the size of the material, the zeta potential, or the administered dose, each of these properties were plotted against the cumulative excretion. The dots were color-coded either for the type of administration (Figures 3B,D) or the material class (Figures 3A,C) to see if there was any relationship. Only material classes or administration types with at least three data points were used. Categories with <3 data points are shown together as “All other” just for illustrative purposes. Not all graphs have the same amount of points as for some data points the specific information was missing. For example, only 96 of the 192 data points have a zeta potential mentioned in the original study, therefore there are only 96 points in the graph for zeta potential and not 192. Plotting all data points together (Figure 3A), it can be seen that most (94%) of the materials are below 200 nm in size, the majority (79%) even below 100 nm, which would be the currently accepted threshold for the nanoparticle definition (European Commission, 2011). Regarding the zeta potential (Figure 3C), the majority (67%) of the data points have a negative zeta potential, only a few (33%) have a positive zeta potential. The doses used in most studies are below 100 mg/kg or even less, only a very small amount of studies used higher doses (Figure 3D). The plots for cumulative excretion versus zeta potential of the nanomaterial color-coded by type of administration and cumulative excretion versus dose of the nanomaterial color-coded by material class can be found in the Supporting Information in Figures S1, S2, respectively.

### Data Evaluation

Several studies report size and surface charge of nanoparticles to be of major influence for their biodistribution and excretion. Small particles (Soo Choi et al., 2007; Semmler-Behnke et al., 2008; Li D. et al., 2016; Jasinski et al., 2018) and positively-charged particles (Alric et al., 2013) are reported to be excreted faster than larger or negatively and neutrally-charged particles. However, looking at the graphs above, there seems to be no correlation between size or zeta potential and excretion neither for different types of administration nor for different material classes. Therefore, a multilinear regression was calculated for the 86 data points for which the size, dose, and zeta potential was available to check if there was any relationship. Size, dose and zeta potential were used as input values and the cumulative excretion of feces and urine in percent as the output. The calculations show that using zeta potential, size, and dose of nanomaterials, the accuracy of predicting the cumulative excretion is low with *R*^2^ being only 0.29. The plot of observed vs. predicted values shown in the Figure S3 reveals that the multilinear regression does not result in an acceptable fit. Taking all data together, it is therefore not possible to predict the amount excreted based on size, zeta potential and amount administered.

Regarding dose dependencies, Xu et al. (2018) have found strong dose-dependent renal clearance of glutathione-coated gold nanoparticles. At higher doses, the same can be seen in the graphs considering all types of nanoparticles. This might be explained by the fact that these doses are so high that the tissues are saturated with the material and the body cannot take up more of the nanomaterial and it is therefore excreted.

**Figure 4:**
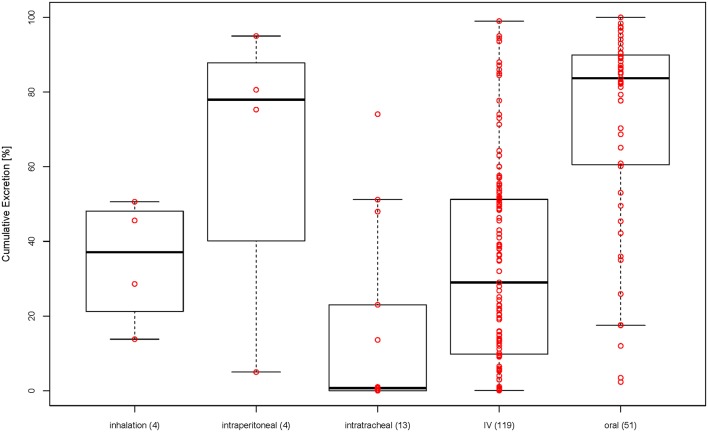
Cumulative excretion for different types of administration (in brackets: number of data points for each type of administration).

Looking at Figure 3B showing the size against excretion color-coded by type of administration, there seems to be a general trend of orally administered particles (blue dots) being excreted more than intravenously administered particles (red dots). Therefore, we have plotted the cumulative excretion vs. the administration for all administration types with three or more data points. Figure 4 shows a boxplot of the cumulative excretion distribution for the five types of administration. The data points are plotted in red circles for each type of administration and the number of data points available for each type of administration is written in brackets next to the administration type.

To test whether the cumulative excretion of the different types of administration is statistically different, we applied a one-way analysis of variance (ANOVA) followed by a *post-hoc* Turkey test on the data set (Table 1). The criterion for statistical significance was *p* < 0.05. We found that only IV-oral, oral-intratracheal, oral-inhalation, and intratracheal-intraperitoneal were significantly different.

**Table 1:** *p*-values from ANOVA for testing statistical difference between different types of administration (*p* < 0.05 in green, *p* > 0.05 in red).

	**Oral**	**Intratracheal**	**Inhalation**	**Intraperitoneal**
IV	<0.001	0.202	0.999	0.154
Oral		<0.001	0.045	0.964
Intratracheal			0.748	0.017
Inhalation				0.524

### Prediction of Excretion for Environmental Risk Assessment

In environmental risk assessments, the potential hazard of a material is compared to the extent the material will come in contact with an organism (ECHA, 2016). Several environmental hazard assessments have been performed on various nanomaterials: Coll et al. (2016) for nano-Ag, CNT, nano-TiO_2_, and nano-ZnO in freshwater; Hauser et al. (2019) for chitosan, nano-chitosan and HAP in freshwater, and chitosan in soil; Mahapatra et al. (2018) for nano-Au in freshwater; Wang and Nowack (2018) for nano-Al_2_O_3_, nano-SiO_2_, nano iron oxides, nano-CeO_2_, and QDs in freshwater. On the other hand, only one study has been performed so far on environmental exposure to NBMs (Mahapatra et al., 2015 for nano-Au). Therefore, more research is needed on the exposure side before environmental risk assessments of NBMs can be performed. As often the NBMs in question are only in the development stage and not yet on the market, the only way to estimate the prospective environmental concentration is through mathematical models (Gottschalk et al., 2009). The amount of a nanomaterial released into a technical or environmental compartment is a central point in any release model (Gottschalk and Nowack, 2011). For NBMs the main relevant release process is the excretion from the human body. If most of the NBM is excreted, it will end up in the wastewater, if it stays in the body or is metabolized, there is no immediate release into water.

**Figure 5:**
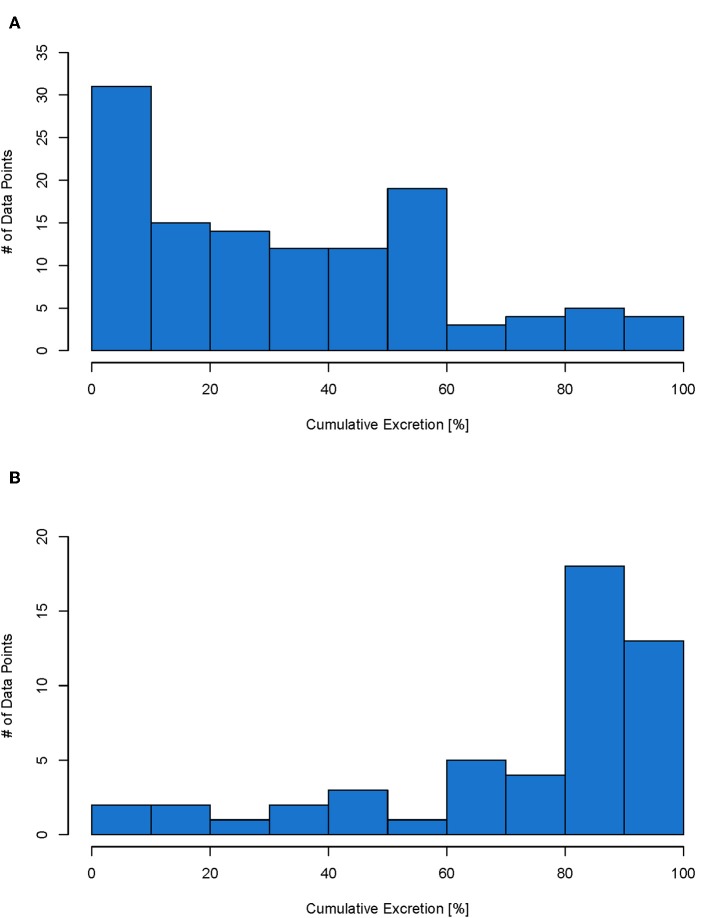
Histogram for IV (total of 114 data points) **(A)** and oral (total of 51 data points) **(B)** administration. Each data point represents the cumulative excretion of a material with specific material properties from one study.

The excretion data collected in the database (Table S1) can be used to predict excretion for a specific NBM or be used to obtain a generic excretion rate for NBM with a specific administration. So if a specific material has its own data, then the real excretion for this material can be used in the model. If however for the material in question, no own data is available, then data from the database can be used in the form of probability distributions. Therefore, for each type of administration, a histogram was prepared to show the distribution of the data points. For IV and oral administration, there are enough data points to see the distribution (see Figures 5A,B below). For inhalation only four data points were available. The histogram for inhalation can be found in the Figure S4. As intratracheal and intraperitoneal administration are not used on humans, their data are not shown here and will not be further evaluated. The distributions shown in Figure 5 represent the probability that a NBM is excreted to a certain extent and can be used as input value to parameterize excretion in probabilistic exposure models such as DPMFA (dynamic probabilistic material flow analysis) (Bornhöft et al., 2016).

Recently published studies have focused on evaluating small difference in particles characteristics and their influence on the biodistribution and excretion. It is generally believed that particles below 5.5 nm in size get rapidly cleared from the body through urinary excretion (Soo Choi et al., 2007). Du et al. (2017) evaluated urinary excretion of sub-nm gold particles with the same surface ligands but different sizes after IV injection. They found that a size reduction of just a few atoms resulted in a decrease in urinary clearance. As in our database, no other materials were in the sub-nm size-range, we could not confirm this on a general basis with other materials. As mentioned before, we have not found a size dependent relationship. Cassano et al. (2019) compared the excretion of silver, gold and platinum nanoparticles and found that while gold nanoparticles are predominantly excreted in urine, silver nanoparticles were almost completely found in feces. We have only analyzed the total excretion, however, it would be interesting to evaluate the route of excretion for the different NBMs. Jasinski et al. (2018) evaluated the effect of shape of RNA nanoparticles on their biodistribution. They compared squared, triangular and pentagon-shaped RNA nanoparticles of 10 nm size. Fluorescent images showed a high fluorescence in kidneys after 12 h for nanosquared, but none for the triangle and very little for the pentagon-shaped nanoparticles. Most studies used round nanoparticles, so to study the general effect of shape, more studies using differently shaped nanoparticles would be needed in the future.

The data collected in the database are all from animal studies. No study is available in which pharmacokinetic profiles for NBMs are compared between animals and humans to get an indication on the extrapolation of animal data to humans with regard to excretion. Data on excretion for other pharmaceuticals are available for different animals and humans. Mamidi et al. (2014) performed an excretion study of orally administered canagliflozin (used for the treatment of type 2 diabetes) in mice, rats, dogs, and humans. They have found a total excretion of canagliflozin and its metabolites of 97.8 and 98.3% for male and female mice, respectively, 96.9 and 98.4% for male and female rats, 99.1% for male dogs, and 92.9% for male humans. Maurer et al. (1983) administered bromocriptine (used for the treatment of Parkinson's disease) orally to mice, rats, monkeys, and humans. They have found a total excretion 94.2% and 101.6% for mice with a dose of 3 and 50 mg/kg, respectively, 83.4% for rats, 101.7% for monkeys, and 88.0% for humans. Comparing these studies, the total excretion from humans is in a similar range as the excretion from the animals included in our database. Therefore, we can assume that the excretion of NBMs in humans would also be in a similar range to animals and thus we can use the calculated excretion profiles for further modeling of NBMs administered to humans.

## Data Availability Statement

The raw data supporting the conclusions of this manuscript will be made available by the authors, without undue reservation, to any qualified researcher.

## Author Contributions

MH collected, prepared, evaluated the input data, created the figures and tables for the manuscript, and wrote the manuscript. BN supervised the study, gave inputs on the data, and contributed to the writing of the manuscript. All authors read and approved the final manuscript.

### Conflict of Interest

The authors declare that the research was conducted in the absence of any commercial or financial relationships that could be construed as a potential conflict of interest.
