# Supplementary material for: Meta-Analysis of Pharmacokinetic Studies of Nanobiomaterials for the Prediction of Excretion Depending on Particle Characteristics
Source: Front Bioeng Biotechnol. 2019 Dec 17;7:405. doi: 10.3389/fbioe.2019.00405 (PMC6927930; doi:10.3389/fbioe.2019.00405)
Supplement: Supplementary file 1 [file Data_Sheet_1.PDF]

Meta-analysis of pharmacokinetic studies of nanobiomaterials for the prediction of excretion depending on particle characteristics

Marina Hauser & Bernd Nowack*

Empa, Swiss Federal Laboratories for Materials Science and Technology, Lerchenfeldstrasse 5, 9014 St. Gallen, Switzerland

*Address correspondence to nowack@empa.ch

Supporting Information

1 Table

4 Figures

Table S1: Database with all data points (abbreviations in footnote)

Material	Class	Size (nm)	Animal	Administration	Zeta Potential (mV)	Dose (mg/kg)	Fecal Excretion Measurement (Days after Administration)	Fecal Excretion (%)	Urinary Excretion Measurement (Days after Administration)	Urinary Excretion (%)	Total Excretion (%)	Ref
SiO2	MetalOxide	15	Rat	oral	-60	500	10	77.25	10	7.65	84.9	(1)
SiO2	MetalOxide	15	Rat	oral	-60	500	10	79.09	10	7.43	86.52	(1)
SiO2	MetalOxide	15	Rat	oral	-60	1000	10	74.6	10	6.73	81.33	(1)
SiO2	MetalOxide	15	Rat	oral	-60	1000	10	75.74	10	7.09	82.83	(1)
SiO2	MetalOxide	89	Rat	oral	-76	500	10	78.52	10	7.62	86.14	(1)
SiO2	MetalOxide	89	Rat	oral	-76	500	10	79.52	10	7.56	87.08	(1)
SiO2	MetalOxide	89	Rat	oral	-76	1000	10	75.08	10	7.11	82.19	(1)
SiO2	MetalOxide	89	Rat	oral	-76	1000	10	76.54	10	7.16	83.7	(1)
NCTD-Chitosan	organic	140	Rat	oral	12.26	5	1	4.5	1	13	17.5	(2)
NCTD-Chitosan	organic	140	Rat	oral	12.26	5	1	0.1	1	17.5	17.6	(2)
ZnO	MetalOxide	29	Rat	intratracheal	23	1	28	50	28	1.18	51.18	(3)
ZnO	MetalOxide	28	Rat	intratracheal	-16.2	1	28	47	28	0.98	47.98	(3)
ZnO	MetalOxide	29	Rat	oral	23	5	7	94	7	0.07	94.07	(3)
ZnO	MetalOxide	28	Rat	oral	-16.2	5	7	95	7	0.12	95.12	(3)
SiO2	MetalOxide	53	Rat	IV	-47.5	20	NA	NA	1	32	32	(4)
Au	Metallic	8.5	Mice	intraperitoneal	-22	7.55	NA	NA	28	5	5	(5)
Au	Metallic	5	Rat	IV	-28.3	0.09	1	0.56	1	0.013	0.573	(6)
Au	Metallic	5	Rat	IV	-19.1	0.07	1	0.27	1	0.0023	0.2723	(6)
Au	Metallic	5	Rat	IV	-10.1	0.14	1	6.5	1	0.0038	6.5038	(6)
Au	Metallic	2.4	Rat	IV	-32	NA	3	5	3	69	74	(7)
TiO2	MetalOxide	20	Rat	IV	-12.5	10	30	12.5	30	41	53.5	(8)
Au	Metallic	1.4	Rat	intratracheal	-20.6	0.0104	NA	NA	1	1	1	(9)
Au	Metallic	5	Rat	intratracheal	-21.1	0.138	NA	NA	1	0.009	0.009	(9)
Au	Metallic	18	Rat	intratracheal	-22.8	0.01	NA	NA	1	0.002	0.002	(9)
Au	Metallic	80	Rat	intratracheal	-22.3	0.0704	NA	NA	1	0.001	0.001	(9)
Au	Metallic	200	Rat	intratracheal	-41.3	0.0564	NA	NA	1	0.006	0.006	(9)

Table S1: cont.

Material	Class	Size (nm)	Animal	Administration	Zeta Potential (mV)	Dose (mg/kg)	Fecal Excretion Measurement (Days after Administration)	Fecal Excretion (%)	Urinary Excretion Measurement (Days after Administration)	Urinary Excretion (%)	Total Excretion (%)	Ref
Au	Metallic	16.1	Rat	IV	-49.4	0.7	5	0.2	NA	NA	0.2	(10)
Au	Metallic	16.3	Rat	IV	-34.3	0.7	5	0.05	NA	NA	0.05	(10)
Au	Metallic	1.4	Rat	IV	-20.6	0.0124	1	4.5	1	5	9.5	(11)
Au	Metallic	5	Rat	IV	-21.1	0.1748	1	0.6	1	0.0125	0.6125	(11)
Au	Metallic	18	Rat	IV	-22.8	0.0116	1	0.6	1	0.003	0.603	(11)
Au	Metallic	80	Rat	IV	-22.3	0.074	1	0.5	1	0.0001	0.5001	(11)
Au	Metallic	200	Rat	IV	-41.3	0.0792	1	0.1	1	0.002	0.102	(11)
Au	Metallic	1.4	Rat	oral	-20.6	0.004	1	82.4	1	0.055	82.455	(12)
Au	Metallic	5	Rat	oral	-21.1	0.1096	1	25.9	1	0.01	25.91	(12)
Au	Metallic	18	Rat	oral	-22.8	0.0096	1	70.3	1	0.01	70.31	(12)
Au	Metallic	80	Rat	oral	-22.3	0.0796	1	77.7	NA	NA	77.7	(12)
Au	Metallic	200	Rat	oral	-41.3	0.076	1	65.1	NA	NA	65.1	(12)
ZnO	MetalOxide	20	Rat	oral	-28.1	50	14	59.75	14	1.13	60.88	(13)
ZnO	MetalOxide	20	Rat	oral	-28.1	300	14	78.54	14	0.78	79.32	(13)
ZnO	MetalOxide	20	Rat	oral	-28.1	2000	14	94.67	14	0.49	95.16	(13)
ZnO	MetalOxide	20	Rat	oral	-28.1	50	14	48.55	14	0.98	49.53	(13)
ZnO	MetalOxide	20	Rat	oral	-28.1	300	14	68.07	14	0.61	68.68	(13)
ZnO	MetalOxide	20	Rat	oral	-28.1	2000	14	96.91	14	0.32	97.23	(13)
ZnO	MetalOxide	70	Rat	oral	-33.3	50	14	58.67	14	1.47	60.14	(13)
ZnO	MetalOxide	70	Rat	oral	-33.3	300	14	76.95	14	0.71	77.66	(13)
ZnO	MetalOxide	70	Rat	oral	-33.3	2000	14	95.85	14	0.5	96.35	(13)
ZnO	MetalOxide	70	Rat	oral	-33.3	50	14	51.66	14	1.36	53.02	(13)
ZnO	MetalOxide	70	Rat	oral	-33.3	300	14	72.95	14	9.61	82.56	(13)
ZnO	MetalOxide	70	Rat	oral	-33.3	2000	14	97.12	14	0.34	97.46	(13)
ZnO	MetalOxide	20	Rat	oral	25	50	14	85.08	14	1.31	86.39	(14)
ZnO	MetalOxide	20	Rat	oral	25	300	14	88.94	14	0.57	89.51	(14)
ZnO	MetalOxide	20	Rat	oral	25	2000	14	90.05	14	0.33	90.38	(14)
ZnO	MetalOxide	20	Rat	oral	25	50	14	83.85	14	1.41	85.26	(14)

Table S1: cont.

Material	Class	Size (nm)	Animal	Administration	Zeta Potential (mV)	Dose (mg/kg)	Fecal Excretion Measurement (Days after Administration)	Fecal Excretion (%)	Urinary Excretion Measurement (Days after Administration)	Urinary Excretion (%)	Total Excretion (%)	Ref
ZnO	MetalOxide	20	Rat	oral	25	300	14	87.6	14	0.56	88.16	(14)
ZnO	MetalOxide	20	Rat	oral	25	2000	14	91.44	14	0.26	91.7	(14)
ZnO	MetalOxide	70	Rat	oral	29.1	50	14	87.87	14	1.33	89.2	(14)
ZnO	MetalOxide	70	Rat	oral	29.1	300	14	88.71	14	0.57	89.28	(14)
ZnO	MetalOxide	70	Rat	oral	29.1	2000	14	92.69	14	0.3	92.99	(14)
ZnO	MetalOxide	70	Rat	oral	29.1	50	14	84.94	14	1.26	86.2	(14)
ZnO	MetalOxide	70	Rat	oral	29.1	300	14	97.82	14	0.57	98.39	(14)
ZnO	MetalOxide	70	Rat	oral	29.1	2000	14	90.3	14	0.25	90.55	(14)
QD	QD	3.5	Mice	IV	-37	3.6	NA	NA	7	0.1	0.1	(15)
Ag	Metallic	7.9	Rat	IV	-17.55	1	1	9	1	0.077	9.077	(16)
Ag	Metallic	7.9	Rat	IV	-17.55	10	1	51.12	1	0.0198	51.1398	(16)
Au	Metallic	2.1	Mice	IV	-48	NA	NA	NA	3	63	63	(17)
Au	Metallic	2.1	Mice	IV	-48	NA	NA	NA	3	51	51	(17)
Au	Metallic	2.3	Mice	IV	-22.1	NA	NA	NA	3	52	52	(17)
Au	Metallic	2.3	Mice	IV	-22.1	NA	NA	NA	3	50	50	(17)
NMOP	organic	40	Mice	IV	-15	5	7	14.5	7	70	84.5	(18)
ZnO	MetalOxide	70	Mice	IV	-30	3.43	1	12.5	1	0.2	12.7	(19)
SiO2	Metallic	150	Rat	IV	-40.5	2	NA	NA	4	36.23	36.23	(20)
CeO2	MetalOxide	31	Rat	IV	-56	100	14	0.407	14	0.0114	0.4184	(21)
CeO2	MetalOxide	6.6	Rat	intratracheal	30.1	2	28	23	NA	NA	23	(22)
CeO2	MetalOxide	6.6	Rat	oral	30.1	5	7	100	NA	NA	100	(22)
SiO2	MetalOxide	120	Mice	IV	-39.4	20	3	8.5	3	13	21.5	(23)
SiO2	MetalOxide	120	Mice	IV	32.4	20	3	5.5	3	14.8	20.3	(23)
SiO2	MetalOxide	136	Mice	IV	-36.6	20	3	15.4	3	22.7	38.1	(23)
SiO2	MetalOxide	136	Mice	IV	36.7	20	3	12.9	3	21.9	34.8	(23)
SiO2	MetalOxide	115	Mice	IV	-50.4	20	3	11.6	3	13.5	25.1	(23)
SiO2	MetalOxide	115	Mice	IV	17	20	3	5.2	3	10.6	15.8	(23)
SLN	organic	160	Rat	oral	-35.43	20	1	34.17	1	1.79	35.96	(24)

Table S1: cont.

Material	Class	Size (nm)	Animal	Administration	Zeta Potential (mV)	Dose (mg/kg)	Fecal Excretion Measurement (Days after Administration)	Fecal Excretion (%)	Urinary Excretion Measurement (Days after Administration)	Urinary Excretion (%)	Total Excretion (%)	Ref
SLN	organic	160	Rat	oral	-35.43	20	1	41.56	1	0.64	42.2	(23)
UCNPs	Other	34.4	Mice	IV	-6.63	NA	1	40.9	1	1.1	42	(25)
Aminoclay	Clay	44	Mice	IV	0.52	1	3	41	3	58	99	(26)
Aminoclay	Clay	44	Mice	oral	0.52	20	3	89	3	0	89	(26)
PLGA	organic	133.5	Mice	IV	-54.2	12	NA	NA	1	15	15	(27)
PLGA	organic	67	Mice	IV	-5.2	12	NA	NA	1	3	3	(27)
SLN	organic	183	Mice	IV	-20	NA	NA	NA	1	29	29	(28)
SLN	organic	182	Mice	IV	-6	NA	NA	NA	1	29	29	(28)
Au	Metallic	17	Swine	IV	-23.2	2	NA	NA	7	10	10	(29)
Au	Metallic	64	Swine	IV	106.2	2	NA	NA	3	3	3	(29)
GBN	Other	1.1	Rat	IV	0	NA	14	4	14	84	88	(30)
TiO2	MetalOxide	25	Rat	IV	20	8.5	NA	NA	7	0.12	0.12	(31)
Gado-6Si-NP	Other	1.6	Rat	IV	6	NA	18	10	18	85	95	(32)
PAA	organic	30.9	Rat	IV	NA	44.6	5	4.1	5	1.2	5.3	(33)
PAA	organic	30.9	Rat	IV	NA	27.6	5	4.9	5	0.5	5.4	(33)
PMM	organic	250	Rat	IV	NA	44.4	6	48	6	38	86	(34)
Au	Metallic	1.4	Rat	intratracheal	NA	NA	1	5	1	8.6	13.6	(35)
Au	Metallic	18	Rat	intratracheal	NA	NA	1	0.5	1	0.1	0.6	(35)
Au	Metallic	2	Mice	IV	NA	40	NA	NA	5	63	63	(36)
Au	Metallic	6	Mice	IV	NA	40	NA	NA	1	4	4	(36)
Au	Metallic	13	Mice	IV	NA	40	NA	NA	1	0.5	0.5	(36)
Au	Metallic	3.5	Mice	IV	NA	NA	NA	NA	1	0.1	0.1	(36)
Au	Metallic	2.1	Mice	intraperitoneal	NA	7.55	NA	NA	28	95	95	(5)
Au	Metallic	2.4	Mice	IV	NA	86.4	1	9.5	1	46	55.5	(37)
Au	Metallic	4.5	Mice	IV	NA	36.92	1	12	1	8.5	20.5	(37)
Au	Metallic	4.7	Mice	IV	NA	39.56	1	20	1	8	28	(37)
Au	Metallic	5	Mice	IV	NA	16	5	8.8	5	37.5	46.3	(38)
Au	Metallic	5	Mice	IV	NA	16	5	2.3	5	12.5	14.8	(38)

Table S1: cont.

Material	Class	Size (nm)	Animal	Administration	Zeta Potential (mV)	Dose (mg/kg)	Fecal Excretion Measurement (Days after Administration)	Fecal Excretion (%)	Urinary Excretion Measurement (Days after Administration)	Urinary Excretion (%)	Total Excretion (%)	Ref
Au	Metallic	11	Mice	IV	NA	16	5	3	5	3	6	(38)
Au	Metallic	22	Mice	IV	NA	16	4	1.98	4	13.98	15.96	(38)
Au	Metallic	22	Mice	IV	NA	16	4	2.09	4	9.95	12.04	(38)
Au	Metallic	5	Mice	IV	NA	16	5	2	5	12	14	(38)
Au	Metallic	2.8	Rat	intratracheal	neg	0.0032	NA	NA	1	0.7	0.7	(9)
Au	Metallic	2.8	Rat	intratracheal	pos	0.078	NA	NA	1	0.3	0.3	(9)
Au	Metallic	2.8	Rat	IV	neg	0.0064	NA	NA	1	0.7	0.7	(11)
Au	Metallic	2.8	Rat	IV	pos	0.116	NA	NA	1	0.2	0.2	(11)
Au	Metallic	2.8	Rat	oral	neg	0.0056	1	45.4	NA	NA	45.4	(12)
Au	Metallic	2.8	Rat	oral	pos	0.0656	1	35	NA	NA	35	(12)
Au	Metallic	2.6	Mice	IV	NA	NA	NA	NA	2	51	51	(39)
Fullerene	Carbon-based	NA	Mice	oral	NA	350	4	97	4	0.2	97.2	(40)
Fullerene	Carbon-based	NA	Mice	IV	NA	350	4	5.4	NA	NA	5.4	(40)
Fullerene	Carbon-based	NA	Rat	IV	NA	NA	5	5.27	5	14.22	19.49	(41)
QD	QD	21.3	Mice	IV	NA	NA	5	33.4	5	23.8	57.2	(42)
Fe2O3	MetalOxide	22	Rat	intratracheal	NA	16	50	73.37	50	0.69	74.06	(43)
Ag	Metallic	2.6	Mice	IV	NA	70	NA	NA	2	51.36	51.36	(44)
Au/Ag	Metallic	2.6	Mice	IV	NA	70	NA	NA	2	52.99	52.99	(44)
Au/Ag	Metallic	2.6	Mice	IV	NA	70	NA	NA	2	48.69	48.69	(44)
Au	Metallic	2.6	Mice	IV	NA	70	NA	NA	2	45.57	45.57	(44)
Au	Metallic	2.5	Mice	IV	NA	1.5	NA	NA	1	39	39	(45)
Au	Metallic	2.5	Mice	IV	NA	5.8	NA	NA	1	39	39	(45)
Au	Metallic	2.5	Mice	IV	NA	10.2	NA	NA	1	39	39	(45)
Au	Metallic	2.5	Mice	IV	NA	15	NA	NA	1	41	41	(45)
Au	Metallic	2.5	Mice	IV	NA	183	NA	NA	1	43	43	(45)
Au	Metallic	2.5	Mice	IV	NA	365	NA	NA	1	50.4	50.4	(45)
Au	Metallic	2.5	Mice	IV	NA	693	NA	NA	1	57.6	57.6	(45)
Au	Metallic	2.5	Mice	IV	NA	1059	NA	NA	1	71.3	71.3	(45)

Table S1: cont.

Material	Class	Size (nm)	Animal	Administration	Zeta Potential (mV)	Dose (mg/kg)	Fecal Excretion Measurement (Days after Administration)	Fecal Excretion (%)	Urinary Excretion Measurement (Days after Administration)	Urinary Excretion (%)	Total Excretion (%)	Ref
Au	Metallic	2.5	Monkey	IV	NA	250	1	1.1	1	54	55.1	(45)
Au	Metallic	2.3	Mice	IV	NA	62.2	NA	NA	1	55	55	(46)
Silica C Dots	QD	3.3	Mice	IV	NA	NA	NA	NA	2	73.04	73.04	(47)
Silica C Dots	QD	6	Mice	IV	NA	NA	NA	NA	2	64.27	64.27	(47)
Silica C Dots	QD	7	Mice	IV	NA	NA	4	15	4	72.2	87.2	(48)
Au	Metallic	40	Mice	IV	NA	NA	7	9	7	14	23	(46)
MSN	MetalOxide	80	Mice	IV	NA	5	NA	NA	30	43	43	(49)
MSN	MetalOxide	120	Mice	IV	NA	5	NA	NA	30	60	60	(49)
MSN	MetalOxide	200	Mice	IV	NA	5	NA	NA	30	51	51	(49)
MSN	MetalOxide	360	Mice	IV	NA	5	NA	NA	30	57	57	(49)
MSN	MetalOxide	80	Mice	IV	NA	5	NA	NA	30	38	38	(49)
MSN	MetalOxide	120	Mice	IV	NA	5	NA	NA	30	35	35	(49)
MSN	MetalOxide	200	Mice	IV	NA	5	NA	NA	30	23	23	(49)
MSN	MetalOxide	360	Mice	IV	NA	5	NA	NA	30	22	22	(49)
MSN	MetalOxide	115	Mice	IV	NA	50	4	21	4	73.4	94.4	(50)
CeO2	MetalOxide	146	Rat	inhalation	NA	23.6	1	12.6	1	1.2	13.8	(51)
CeO2	MetalOxide	195	Rat	inhalation	NA	122.7	1	28.5	1	0.1	28.6	(51)
CeO2	MetalOxide	174	Rat	inhalation	NA	66.2	1	45.5	1	0.1	45.6	(51)
CeO2	MetalOxide	151	Rat	inhalation	NA	60.0	1	47.5	1	3.1	50.6	(51)
Isobutyl cyanoacrylate	organic	254	Mice	IV	NA	NA	7	27	7	66.6	93.6	(52)
PCL	organic	100	Rat	oral	NA	5	2	1.5	2	0.83	2.33	(53)
PCL	organic	160	Rat	oral	NA	5	2	2.8	2	0.67	3.47	(53)
MSN	MetalOxide	115	Mice	intraperitoneal	NA	50	6	36.6	6	44	80.6	(54)
MSN	MetalOxide	115	Mice	intraperitoneal	NA	50	6	28	6	47.3	75.3	(54)
PLA	organic	170	Rat	IV	NA	17.2	7	0.68	7	11.2	11.88	(55)
SiO2	MetalOxide	20	Rat	IV	NA	10	30	9.2	30	30	39.2	(56)
SiO2	MetalOxide	80	Rat	IV	NA	10	30	10.8	30	38	48.8	(56)
PAMAM	organic	5	Mice	IV	NA	7	7	2.96	7	35.7	38.66	(57)

Table S1: cont.

Material	Class	Size (nm)	Animal	Administration	Zeta Potential (mV)	Dose (mg/kg)	Fecal Excretion Measurement (Days after Administration)	Fecal Excretion (%)	Urinary Excretion Measurement (Days after Administration)	Urinary Excretion (%)	Total Excretion (%)	Ref
PAMAM	organic	5	Mice	IV	NA	3.52	7	5.41	7	48.7	54.11	(57)
Au	Metallic	25	Rat	IV	NA	NA	10	1.03	10	1.9	2.93	(58)
Au	Metallic	25	Rat	oral	NA	NA	10	56.46	10	3.6	60.06	(58)
PBN	organic	161.2	Rat	IV	NA	NA	NA	NA	2	12	12	(59)
Chitosan	organic	NA	Rat	IV	NA	3.3	3	4.86	3	4.27	9.13	(60)
Chitosan	organic	NA	Rat	intrahepatic	NA	33.3	3	0.54	3	0.53	1.07	(60)
Au	Metallic	1.2	Mice	IV	NA	NA	NA	NA	28	43.6	43.6	(61)
Au	Metallic	1.2	Mice	IV	NA	NA	NA	NA	28	77.7	77.7	(61)
Au	Metallic	1.2	Mice	IV	NA	NA	NA	NA	28	49.5	49.5	(61)
Au	Metallic	1.2	Mice	IV	NA	NA	NA	NA	28	48.4	48.4	(61)
Au	Metallic	1.2	Mice	IV	NA	NA	NA	NA	28	53	53	(61)
TMDC	Other	52	Mice	IV	NA	10	30	12.3	30	24.22	36.52	(62)
TMDC	Other	43	Mice	IV	NA	10	30	4.1	30	5.45	9.55	(62)
TMDC	Other	59	Mice	IV	NA	10	30	2.3	30	7	9.3	(62)
PMMA	organic	9.7	Rat	IV	NA	0.09	2	11.8	2	12.5	24.3	(63)
PMMA	organic	17	Rat	IV	NA	0.09	2	12.4	2	10.4	22.8	(63)
PMMA	organic	20	Rat	IV	NA	0.09	2	8.8	2	5.1	13.9	(63)
MWCNT	Carbon-based	25	Mice	IV	NA	30	1	9.701	1	1.51	11.211	(64)
MWCNT	Carbon-based	25	Mice	IV	NA	30	1	4.42	1	1.269	5.689	(64)
NDs	Carbon-based	217	Mice	IV	NA	30	1	11.477	1	1.821	13.298	(64)
NDs	Carbon-based	217	Mice	IV	NA	30	1	10.998	1	2.54	13.538	(64)
Ag_2S	Metallic	3.1	Mice	IV	-11	250	1	0.2	1	84.8	85	(65)
Au	Metallic	1	Mice	IV	NA	NA	NA	NA	1	51.57	51.57	(66)
Au	Metallic	0.7	Mice	IV	NA	NA	NA	NA	1	26.82	26.82	(66)
Au	Metallic	0.6	Mice	IV	NA	NA	NA	NA	1	22.9	22.9	(66)
Au	Metallic	0.4	Mice	IV	NA	NA	NA	NA	1	19.07	19.07	(66)

Table S1: cont.

GBN: Gadolinium-based nanoparticle
MSN: mesomorphous silica nanoparticle
MWCNT: multi-walled carbon nanotubes
NCTD-Chitosan: norcantharidin chitosan
ND: nanodiamonds
NMOP: nanoscale metal organic particle
PAA: polyacrylamide
PAMAM: poly(amidoamine)
PBN: polybutylcyanoacrylate
PCL: Polycaprolacetone
PLA: polylactic acid
PLGA: poly(lactic-glycolic acid)
PMM: Poly(methylidene malonate)
PMMA: poly(methyl methacrylate)
SLN: solid lipid nanoparticle
TMDC: transition metal dichalcogenide
UCNPs: Upconverting nanoparticles

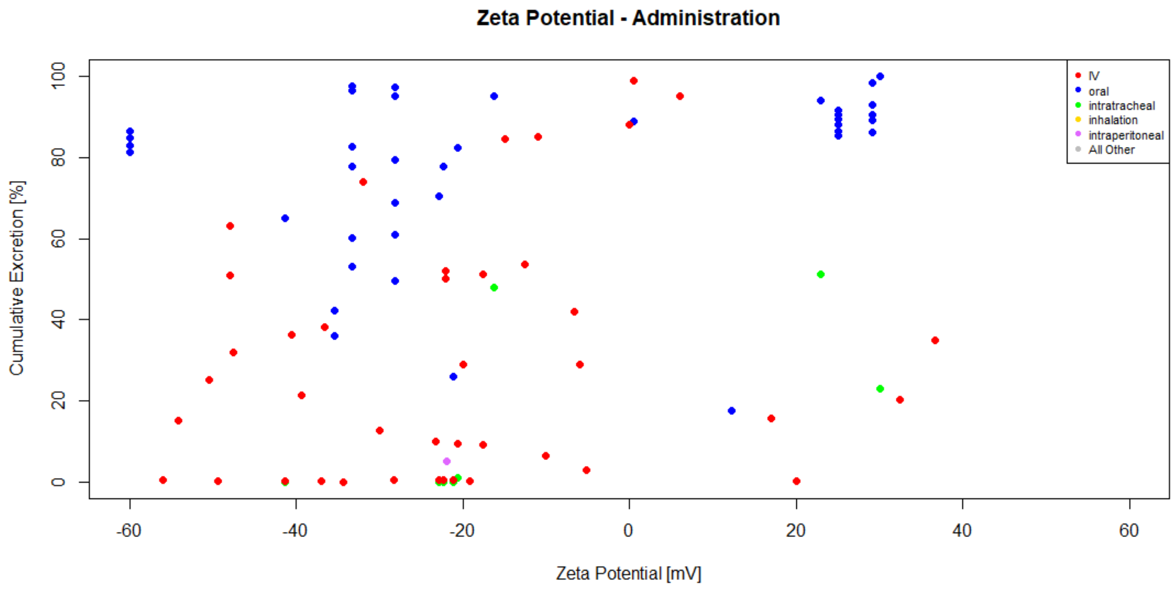

Figure S1: Cumulative excretion versus zeta potential of the nanomaterial color-coded by type of administration

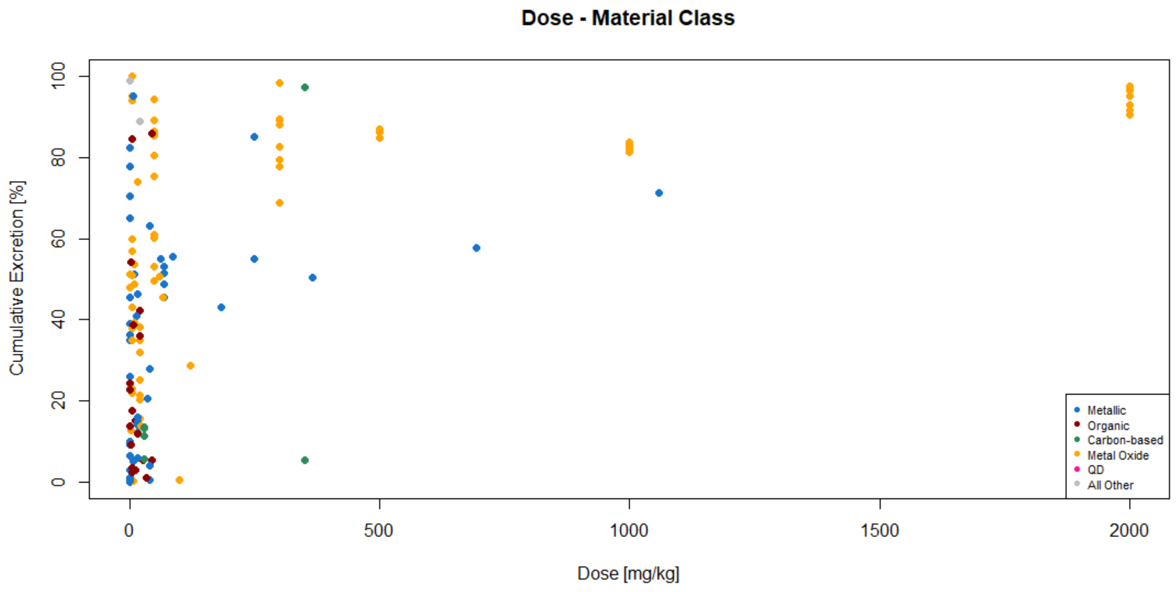

Figure S2: Cumulative excretion versus dose of the nanomaterial color-coded by material class

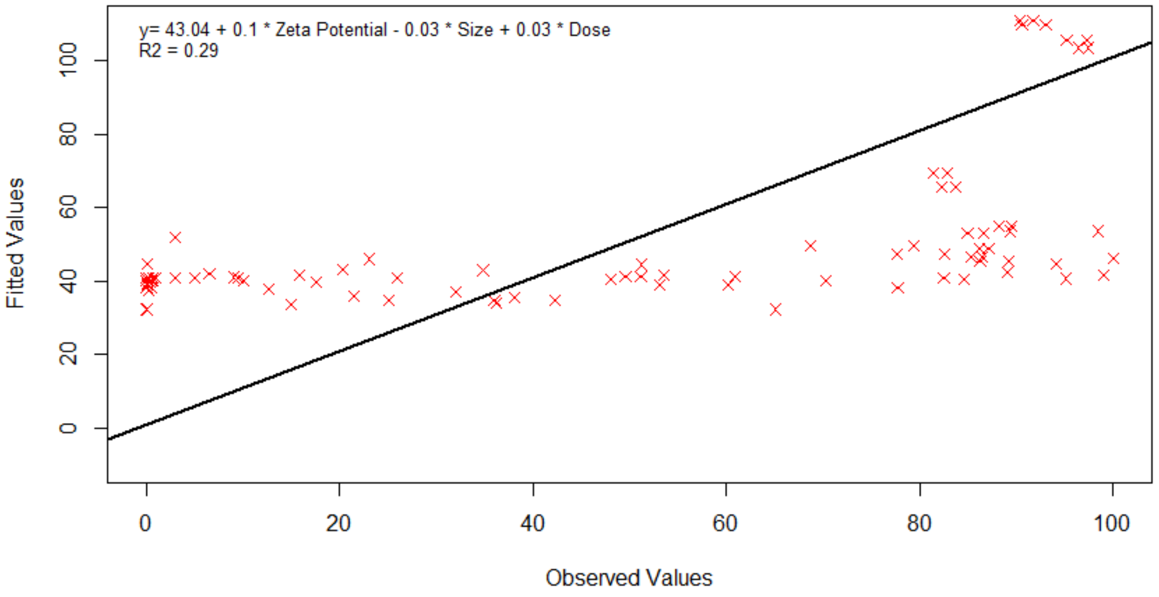

Figure S3: Observed values vs values fitted by multilinear regression using zeta potential, size and dose as input values and cumulative excretion as output

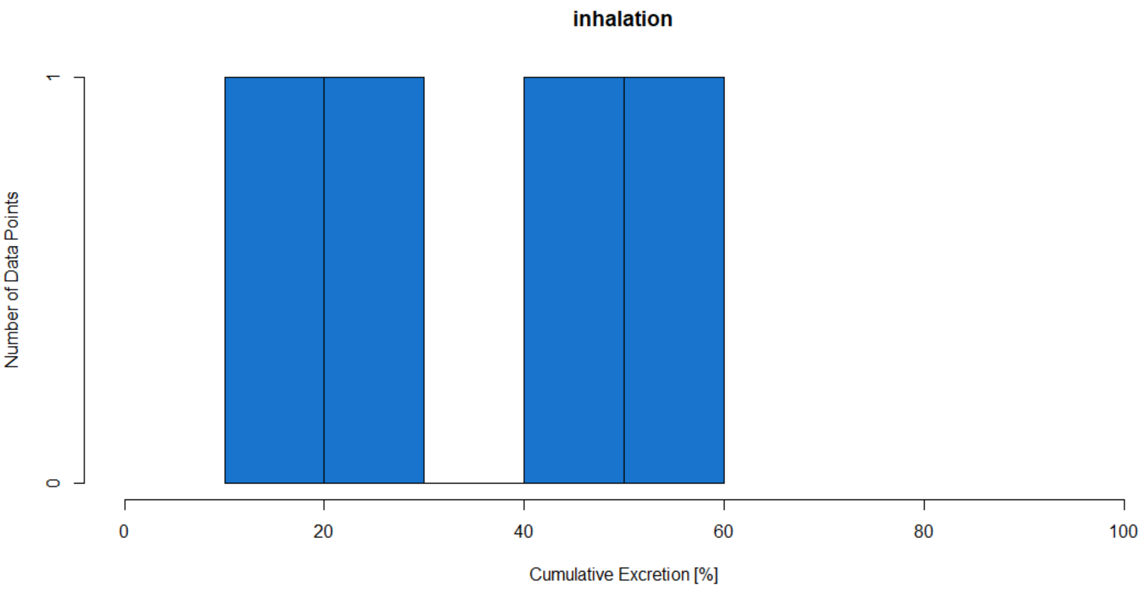

Figure S4: Histogram for inhalation (total of four data points)

References

1. Lee J-A, Kim M-K, Paek H-J, Kim Y-R, Kim M-K, Lee J-K, et al. Tissue distribution and excretion kinetics of orally administered silica nanoparticles in rats. Int J Nanomedicine. 2014;251.

2. Ding XY, Hong CJ, Liu Y, Gu ZL, Xing KL, Zhu AJ, et al. Pharmacokinetics, tissue distribution, and metabolites of a polyvinylpyrrolidonecoated norcantharidin chitosan nanoparticle formulation in rats and mice, using LC-MS/MS. Int J Nanomedicine. 2012;7:1723–35.

3. Konduru N V., Murdaugh KM, Sotiriou GA, Donaghey TC, Demokritou P, Brain JD, et al. Bioavailability, distribution and clearance of tracheally-instilled and gavaged uncoated or silica-coated zinc oxide nanoparticles. Part Fibre Toxicol. 2014;11(1):1–13.

4. Waegeneers N, Brasseur A, Van Doren E, Van der Heyden S, Serreyn PJ, Pussemier L, et al. Short-term biodistribution and clearance of intravenously administered silica nanoparticles. Toxicol Reports [Internet]. 2018;5(April 2017):632–8. Available from: https://doi.org/10.1016/j.toxrep.2018.05.004

5. Zhang XD, Wu D, Shen X, Liu PX, Fan FY, Fan SJ. In vivo renal clearance, biodistribution, toxicity of gold nanoclusters. Biomaterials. 2012;33(18):4628–38.

6. Lipka J, Semmler-Behnke M, Sperling RA, Wenk A, Takenaka S, Schleh C, et al. Biodistribution of PEG-modified gold nanoparticles following intratracheal instillation and intravenous injection. Biomaterials [Internet]. 2010;31(25):6574–81. Available from: http://dx.doi.org/10.1016/j.biomaterials.2010.05.009

7. Alric C, Miladi I, Kryza D, Taleb J, Lux F, Bazzi R, et al. The biodistribution of gold nanoparticles designed for renal clearance. Nanoscale. 2013;5(13):5930–9.

8. Xie G, Sun J, ZhonG. G, Shi L, ZhanG. D. Biodistribution and toxicity of intravenously administered silica nanoparticles in mice. Arch Toxicol. 2010;84(3):183–90.

9. Kreyling WG, Hirn S, Möller W, Schleh C, Wenk A, Celik G, et al. Air-blood barrier translocation of tracheally instilled gold nanoparticles inversely depends on particle size. ACS Nano. 2014;8(1):222–33.

10. Fraga S, Brandão A, Soares ME, Morais T, Duarte JA, Pereira L, et al. Short- and long-term distribution and toxicity of gold nanoparticles in the rat after a single-dose intravenous administration. Nanomedicine Nanotechnology, Biol Med [Internet]. 2014;10(8):1757–66. Available from: http://dx.doi.org/10.1016/j.nano.2014.06.005

11. Hirn S, Semmler-Behnke M, Schleh C, Wenk A, Lipka J, Schäffler M, et al. Particle size-dependent and surface charge-dependent biodistribution of gold nanoparticles after intravenous administration. Eur J Pharm Biopharm [Internet]. 2011;77(3):407–16. Available from: http://dx.doi.org/10.1016/j.ejpb.2010.12.029

12. Schleh C, Semmler-Behnke M, Lipka J, Wenk A, Hirn S, Schäffler M, et al. Size and surface charge of gold nanoparticles determine absorption across intestinal barriers and accumulation in secondary target organs after oral administration. Nanotoxicology.

2012;6(1):36–46.

13. Baek M, Chung HE, Yu J, Lee JA, Kim TH, Oh JM, et al. Pharmacokinetics, tissue distribution, and excretion of zinc oxide nanoparticles. Int J Nanomedicine. 2012;7:3081–97.

14. Paek HJ, Lee YJ, Chung HE, Yoo NH, Lee JA, Kim MK, et al. Modulation of the pharmacokinetics of zinc oxide nanoparticles and their fates in vivo. Nanoscale. 2013;5(23):11416–27.

15. Liang X, Wang H, Grice JE, Li L, Liu X, Xu ZP, et al. Physiologically Based Pharmacokinetic Model for Long-Circulating Inorganic Nanoparticles. Nano Lett. 2016;16(2):939–45.

16. Park EJ, Bae E, Yi J, Kim Y, Choi K, Lee SH, et al. Repeated-dose toxicity and inflammatory responses in mice by oral administration of silver nanoparticles. Environ Toxicol Pharmacol [Internet]. 2010;30(2):162–8. Available from: http://dx.doi.org/10.1016/j.etap.2010.05.004

17. Yu M, Zhou C, Liu L, Zhang S, Sun S, Hankins JD, et al. Interactions of Renal-Clearable Gold Nanoparticles with Tumor Microenvironments: Vasculature and Acidity Effects. Angew Chemie - Int Ed. 2017;56(15):4314–9.

18. Yang Y, Liu J, Liang C, Feng L, Fu T, Dong Z, et al. Nanoscale Metal-Organic Particles with Rapid Clearance for Magnetic Resonance Imaging-Guided Photothermal Therapy. ACS Nano. 2016;10(2):2774–81.

19. Chen JK, Shih MH, Peir JJ, Liu CH, Chou FI, Lai WH, et al. The use of radioactive zinc oxide nanoparticles in determination of their tissue concentrations following intravenous administration in mice. Analyst. 2010;135(7):1742–6.

20. Borak B, Prescha A, Baszczuk A, Pluta J. In Vivo Study on the Biodistribution of Silica Particles. Adv Clin Exp Med. 2012;13–8.

21. Yokel RA, Au TC, MacPhail R, Hardas SS, Butterfield DA, Sultana R, et al. Distribution, elimination, and biopersistence to 90 days of a systemically introduced 30 nm ceria-engineered nanomaterial in rats. Toxicol Sci. 2012;127(1):256–68.

22. He X, Zhang H, Ma Y, Bai W, Zhang Z, Lu K, et al. Lung deposition and extrapulmonary translocation of nano-ceria after intratracheal instillation. Nanotechnology. 2010;21(28):0–8.

23. Yu T, Hubbard D, Ray A, Ghandehari H. In vivo biodistribution and pharmacokinetics of silica nanoparticles as a function of geometry, porosity and surface characteristics. J Control Release [Internet]. 2012;163(1):46–54. Available from: http://dx.doi.org/10.1016/j.jconrel.2012.05.046

24. Armand L, Dagouassat M, Belade E, Simon-deckers A, Le Gouvello S, Tharabat C, et al. Leica ES2 Leica EZ4 Leica EZ4 HD Manual. Part Fibre Toxicol [Internet]. 2013;9(1):1–4. Available from: http://www.mdpi.com/2079-9721/2/3/260/htm%5Cnhttp://www.ncbi.nlm.nih.gov/pubmed/9652404%5Cnhttp://dx.doi.org/10.1016/j.addr.2011.09.001%5Cnhttp://www.pubmedcentral.nih.gov/articlerender.fcgi?artid=4215823&tool=pmcentrez&rendertype=abstract%5Cnhttp://www.j

25. Seo HJ, Nam SH, Im HJ, Park JY, Lee JY, Yoo B, et al. Rapid Hepatobiliary Excretion of Micelle-Encapsulated/Radiolabeled Upconverting Nanoparticles as an Integrated Form. Sci Rep [Internet]. 2015;5(June):1–12. Available from: http://dx.doi.org/10.1038/srep15685

26. Yang L, Ge X, Wan C, Yu F, Li Y. Progress and perspectives in converting biogas to transportation fuels. Renew Sustain Energy Rev [Internet]. 2014;40:1133–52. Available from: http://dx.doi.org/10.1016/j.rser.2014.08.008

27. Avgoustakis K, Beletsi A, Panagi Z, Klepetsanis P, Karydas AG, Ithakissios DS. PLGA-mPEG nanoparticles of cisplatin: In vitro nanoparticle degradation, in vitro drug release and in vivo drug residence in blood properties. J Control Release. 2002;79(1–3):123–35.

28. Heiati H, Tawashi R, Phillips NC. Solid lipid nanoparticles as drug carriers. II. Plasma stability and biodistribution of solid lipid nanoparticles containing the lipophilic prodrug 3′-azido-3′-deoxythymidine palmitate in mice. Int J Pharm. 1998;174(1–2):71–80.

29. Fent GM, Casteel SW, Kim DY, Kannan R, Katti K, Chanda N, et al. Biodistribution of maltose and gum arabic hybrid gold nanoparticles after intravenous injection in juvenile swine. Nanomedicine Nanotechnology, Biol Med [Internet]. 2009;5(2):128–35. Available from: http://dx.doi.org/10.1016/j.nano.2009.01.007

30. Miladi I, Duc G Le, Kryza D, Berniard A, Mowat P, Roux S, et al. Biodistribution of ultra small gadolinium-based nanoparticles as theranostic agent: Application to brain tumors. J Biomater Appl. 2013;28(3):385–94.

31. Elgrabli D, Beaudouin R, Jbilou N, Floriani M, Pery A, Rogerieux F, et al. Biodistribution and clearance of TiO<inf>2</inf> nanoparticles in rats after intravenous injection. PLoS One [Internet]. 2015;10(4):1–13. Available from: http://dx.doi.org/10.1371/journal.pone.0124490

32. Kryza D, Taleb J, Janier M, Marmuse L, Miladi I, Bonazza P, et al. Biodistribution study of nanometric hybrid gadolinium oxide particles as a multimodal SPECT/MR/optical imaging and theragnostic agent. Bioconjug Chem. 2011;22(6):1145–52.

33. Wenger Y, Schneider RJ, Reddy GR, Kopelman R, Jolliet O, Philbert MA. Tissue distribution and pharmacokinetics of stable polyacrylamide nanoparticles following intravenous injection in the rat. Toxicol Appl Pharmacol [Internet]. 2011;251(3):181–90. Available from: http://dx.doi.org/10.1016/j.taap.2010.11.017

34. Lescure F, Seguin C, Breton P, Bourrinet P, Roy D, Couvreur P. Preparation and characterization of novel poly(methylidene malonate 2.1.2.)-made NPs. Pharmaceutical Research, Vol. 11, No. 9; 1994. p. 1270–7.

35. Semmler-Behnke M, Kreyling WG, Lipka J, Fertsch S, Wenk A, Takenaka S, et al. Biodistribution of 1.4- and 18-nm Gold Particles in Rats. Small [Internet]. 2008;4(12):2108–11. Available from: http://doi.wiley.com/10.1002/smll.200800922

36. Zhou C, Long M, Qin Y, Sun X, Zheng J. Luminescent Gold Nanoparticles with Efficient Renal Clearance. NIH Public Access. 2011;29(10):1883–9.

37. Wong OA, Hansen RJ, Ni TW, Heinecke CL, Compel WS, Gustafson DL, et al. Structure–

activity relationships for biodistribution, pharmacokinetics, and excretion of atomically precise nanoclusters in a murine model. Nanoscale [Internet]. 2013;5(21):10525. Available from: http://xlink.rsc.org/?DOI=c3nr03121g

38. Balogh L, Nigavekar SS, Nair BM, Lesniak W, Zhang C, Sung LY, et al. Significant effect of size on the in vivo biodistribution of gold composite nanodevices in mouse tumor models. Nanomedicine Nanotechnology, Biol Med. 2007;3(4):281–96.

39. Zhou C, Hao G, Thomas P, Liu J, Yu M, Sun S, et al. Near-infrared emitting radioactive gold nanoparticles with molecular pharmacokinetics. Angew Chemie - Int Ed. 2012;51(40):10118–22.

40. Yamago S, Tokuyama H, Nakamura E, Kikuchi K, Kananishl S, Sueki K, et al. In viva biological behavior of a water-miscible fullerene: absorption , distribution , excretion and acute toxicity. Curr Biol. 1995;385–9.

41. Cagle DW, Kennel SJ, Mirzadeh S, Alford JM, Wilson LJ. In vivo studies of fullerene-based materials using endohedral metallofullerene radiotracers. Proc Natl Acad Sci U S A. 1999;96(9):5182–7.

42. Chen Z, Chen H, Meng H, Xing G, Gao X, Sun B, et al. Bio-distribution and metabolic paths of silica coated CdSeS quantum dots. Toxicol Appl Pharmacol. 2008;230(3):364–71.

43. Zhu MT, Feng WY, Wang Y, Wang B, Wang M, Ouyang H, et al. Particokinetics and extrapulmonary translocation of intratracheally instilled ferric oxide nanoparticles in rats and the potential health risk assessment. Toxicol Sci. 2009;107(2):342–51.

44. Tang S, Peng C, Xu J, Du B, Wang Q, Vinluan RD, et al. Tailoring Renal Clearance and Tumor Targeting of Ultrasmall Metal Nanoparticles with Particle Density. Angew Chemie - Int Ed. 2016;55(52):16039–43.

45. Xu J, Yu M, Peng C, Carter P, Tian J, Ning X, et al. Dose Dependencies and Biocompatibility of Renal Clearable Gold Nanoparticles: From Mice to Non-human Primates. Angew Chemie - Int Ed. 2018;57(1):266–71.

46. Liu CP, Lin FS, Chien C Te, Tseng SY, Luo CW, Chen CH, et al. In-situ formation and assembly of gold nanoparticles by gum Arabic as efficient photothermal agent for killing cancer cells. Macromol Biosci. 2013;13(10):1314–20.

47. Burns AA, Vider J, Ow H, Herz E, Penate-medina O, Baumgart M, et al. Fluorescent Silica Nanoparticles with. Nano. 2009;

48. Benezra M, Penate-medina O, Zanzonico PB, Schaer D, Ow H, Burns A, et al. Multimodal silica nanoparticles are effective cancer-targeted probes in a model of human melanoma Find the latest version: Technical advance Multimodal silica nanoparticles are effective cancer-targeted probes in a model of human melanoma. J Clin Invest. 2011;121(7):2768–80.

49. He Q, Zhang Z, Gao F, Li Y, Shi J. In vivo biodistribution and urinary excretion of mesoporous silica nanoparticles: Effects of particle size and PEGylation. Small. 2011;7(2):271–80.

50. Lu J, Liong M, Li Z, Zink JI, Tamanoi F. Biocompatibility, biodistribution, and drug-delivery efficiency of mesoporous silica nanoparticles for cancer therapy in animals. Small. 2010;6(16):1794–805.

51. Li D, Morishita M, Wagner JG, Fatouraie M, Wooldridge M, Eagle WE, et al. In vivo biodistribution and physiologically based pharmacokinetic modeling of inhaled fresh and aged cerium oxide nanoparticles in rats. Part Fibre Toxicol [Internet]. 2016;13(1). Available from: http://dx.doi.org/10.1186/s12989-016-0156-2

52. Grislain L, Speiser P, Roland M, Deprez-Decampeneere D, Lenaerts V, Couvreur P. Pharmacokinetics and distribution of a biodegradable drug-carrier. Int J Pharm. 2002;15(3):335–45.

53. Saez A, Guzma M, Molpeceres J, Aberturas MR. Freeze-drying of polycaprolactone and poly(d,l-lactic-glycolic) nanoparticles induce minor particle size changes affecting the oral pharmacokinetics of loaded drugs. In Vivo (Brooklyn). 2000;50:379–87.

54. Lu J, Li Z, Zink JI, Tamanoi F. In vivo tumor suppression efficacy of mesoporous silica nanoparticles-based drug-delivery system: Enhanced efficacy by folate modification. Nanomedicine Nanotechnology, Biol Med [Internet]. 2012;8(2):212–20. Available from: http://dx.doi.org/10.1016/j.nano.2011.06.002

55. Bazile D V, Ropert C, Huve P, Verrecchia T, Marlard M, Frydman A, et al. Body distribution of fully biodegradable [C-13] Poly(lactic acid) nanoparticles coated with albumin. Biomaterials. 1992;13(15):1093–102.

56. Xie G, Sun J, Zhong G. Tissular localization and excretion of intravenously administered silica nanoparticles of different sizes. J Nanoparticle Res. 2012;14(1).

57. Nigavekar SS, Sung LY, Llanes M, El-jawahri A, Lawrence TS, Becker CW, et al. 3H Dendrimer Nanoparticle Organ/Tumor Distribution. Pharm Res. 2004;21(3):476–83.

58. Bednarski M, Dudek M, Knutelska J, Nowiński L, Sapa J, Zygmunt M, et al. The influence of the route of administration of gold nanoparticles on their tissue distribution and basic biochemical parameters: In vivo studies. Pharmacol Reports. 2015;67(3):405–9.

59. Kante B, Couvreur P, Lenaerts V, Guiot P, Roland M, Baudhuin P, et al. Tissue distribution of [3H]actinomycin D adsorbed on polybutylcyanoacrylate nanoparticles. Int J Pharm. 1980;7(1):45–53.

60. Suzuki YS, Momose Y, Higashi N, Shigematsu a, Park KB, Kim YM, et al. Biodistribution and kinetics of holmium-166-chitosan complex (DW-166HC) in rats and mice. J Nucl Med [Internet]. 1998;39(12):2161–6. Available from: http://www.ncbi.nlm.nih.gov/pubmed/9867162

61. Simpson CA, Salleng KJ, Cliffel DE, Feldheim DL. In vivo toxicity, biodistribution, and clearance of glutathione-coated gold nanoparticles. Nanomedicine Nanotechnology, Biol Med [Internet]. 2013;9(2):257–63. Available from: http://dx.doi.org/10.1016/j.nano.2012.06.002

62. Hao J, Song G, Liu T, Yi X, Yang K, Cheng L, et al. In Vivo Long-Term Biodistribution, Excretion, and Toxicology of PEGylated Transition-Metal Dichalcogenides MS2 (M = Mo,

W, Ti) Nanosheets. Adv Sci. 2017;4(1).

63. Pressly ED, Rossin R, Hagooly A, Fukukawa KI, Messmore BW, Welch MJ, et al. Structural effects on the biodistribution and positron emission tomograpgy (PET) imaging of well-defined 64Cu-labeled nanoparticles comprised of amphiphilic block graft copolymers. Biomacromolecules. 2007;8(10):3126–34.

64. Wei Q, Zhan L, Juanjuan B, Jing W, Jianjun W, Taoli S, et al. Biodistribution of co-exposure to multi-walled carbon nanotubes and nanodiamonds in mice. Nanoscale Res Lett. 2012;7(1):473.

65. Hsu JC, Cruz ED, Lau KC, Bouché M, Kim J, Maidment ADA, et al. Renally Excretable and Size-Tunable Silver Sulfide Nanoparticles for Dual-Energy Mammography or Computed Tomography. Chem Mater. 2019;31(19):7845–54.

66. Du B, Jiang X, Das A, Zhou Q, Yu M, Jin R, et al. Glomerular barrier behaves as an atomically precise bandpass filter in a sub-nanometre regime. Nat Nanotechnol. 2017;12(11):1096–102.
